# Synaptic Reorganization in the Adult Rat's Ventral Cochlear Nucleus following Its Total Sensory Deafferentation

**DOI:** 10.1371/journal.pone.0023686

**Published:** 2011-08-22

**Authors:** Heika Hildebrandt, Nadine A. Hoffmann, Robert-Benjamin Illing

**Affiliations:** Neurobiological Research Laboratory, Department of Otorhinolaryngology, University of Freiburg, Freiburg, Germany; The Research Center of Neurobiology-Neurophysiology of Marseille, France

## Abstract

Ablation of a cochlea causes total sensory deafferentation of the cochlear nucleus in the brainstem, providing a model to investigate nervous degeneration and formation of new synaptic contacts in the adult brain. In a quantitative electron microscopical study on the plasticity of the central auditory system of the Wistar rat, we first determined what fraction of the total number of synaptic contact zones (SCZs) in the anteroventral cochlear nucleus (AVCN) is attributable to primary sensory innervation and how many synapses remain after total unilateral cochlear ablation. Second, we attempted to identify the potential for a deafferentation-dependent synaptogenesis. SCZs were ultrastructurally identified before and after deafferentation in tissue treated for ethanolic phosphotungstic acid (EPTA) staining. This was combined with pre-embedding immunocytochemistry for gephyrin identifying inhibitory SCZs, the growth-associated protein GAP-43, glutamate, and choline acetyltransferase. A stereological analysis of EPTA stained sections revealed 1.11±0.09 (S.E.M.)×10^9^ SCZs per mm^3^ of AVCN tissue. Within 7 days of deafferentation, this number was down by 46%. Excitatory and inhibitory synapses were differentially affected on the side of deafferentation. Excitatory synapses were quickly reduced and then began to increase in number again, necessarily being complemented from sources other than cochlear neurons, while inhibitory synapses were reduced more slowly and continuously. The result was a transient rise of the relative fraction of inhibitory synapses with a decline below original levels thereafter. Synaptogenesis was inferred by the emergence of morphologically immature SCZs that were consistently associated with GAP-43 immunoreactivity. SCZs of this type were estimated to make up a fraction of close to 30% of the total synaptic population present by ten weeks after sensory deafferentation. In conclusion, there appears to be a substantial potential for network reorganization and synaptogenesis in the auditory brainstem after loss of hearing, even in the adult brain.

## Introduction

The young adult rat spiral ganglion contains about 16,000 neurons [1] that project to neurons in the cochlear nucleus [2]. The cochlear nucleus is subdivided into a ventral (VCN) and a dorsal (DCN) part. Smaller presynaptic endings are formed in DCN while large to very large endings predominate in VCN [3], including conspicuously large presynaptic specializations known as endbulbs of Held [4].

Apart from primary sensory axons, the cochlear nucleus receives afferents from other auditory sources, among them the contralateral cochlear nucleus [5], the superior olivary complex [6], and the auditory cortex [7], but also from non-auditory regions [8]–[10]. There exists an extended intrinsic circuitry in the cochlear nuclear complex [11]–[13].

Following total primary deafferentation by ablating the cochlea, Gentschev and Sotelo [14] observed in an ultrastructural study that some presynaptic endings change in a ‘clear’ pattern and others in an ‘electron-dense’ pattern, both classified by the authors as indicative of degeneration. Denervated postsynaptic sites were seen to be in part reoccupied by intact axon terminals, a response interpreted to be due to a sliding process of nearby intact terminals rather than to axonal collateral sprouting. More often, though, neurons appeared to be cleared of the free postsynaptic sites by engulfment of the postsynaptic specialization into the cytoplasm. These authors suggest that this prevents induction of new but unspecific synaptic contacts. A loss of nerve cells has not been reported for the adult anteroventral cochlear nucleus (AVCN) after cochlear ablation [15]; apoptotic markers show up only in microglial cells [16].

It remained to be determined whether the physiological silencing of neurons in VCN, but not in DCN, after cochlear ablation [17] is due to the loss of an overwhelming majority of primary sensory synapses, or to a lack of neurons driving network activity. The higher the fraction of primary sensory afferents among all incoming synaptic contacts, the more likely it would seem that VCN is a mere relay along the ascending auditory pathway. If this fraction is small instead, VCN would seem to be involved in complex signal integration. Network modeling relies on knowledge of how many interneurons exist in relation to input and output channels, and what numerical relationship exists between excitatory and inhibitory synapses.

We launched this study by asking two questions. The first concerned the fraction of primary sensory afferent from the cochlea among all synaptic contacts present in VCN. The second question focused on the possibility of a constructive response of the neuronal network to the sudden and, as was expected from axonal tracing studies, massive loss of synapses. To this end, we also aimed to determine the fraction of excitatory, inhibitory, and nascent synaptic contacts before and after deafferentation, employing the growth-associated protein GAP-43 [18], [19] as a marker for axonal and synaptic growth. Combining classical with modern electron microscopical staining techniques, we were able to find conclusive answers to both issues raised.

Understanding the logic behind the interdependence of weakening, loss, regain, and compensational supply of synapses in a nervous network following damage to sense organs or the brain, will provide fundamental knowledge required to properly diagnose patients, develop accurate prognoses for them, and, in the long run, design pinpoint therapies to boost specific dynamic responses over others.

## Methods

### Ethics statement

Care and use of the animals as reported here were approved by the appropriate local agency (Regierungspräsidium Freiburg, Germany, permission number 35/9185.81/G-07/22).

### Animals

This study is based on brain tissue obtained from 37 adult Wistar rats aged 7 to 20 weeks. For cochlear ablation and tracer injection animals were anesthetized with a mixture of S-ketamine (50 mg/kg, Ketanest, Parke-Davis, Ann Arbor, MI, USA) and Xylazine (5 mg/kg, Rompun, Bayer-Leverkusen, Leverkusen, Germany) injected intraperitoneally (i.p.).

### Cochlear ablation

Following a retroauricular incision [20], the bulla tympani was approached and the facial nerve cut upon its exit from the skull. The bulla was opened starting from the outer ear canal and working caudally until the bulging cochlea was visible. The bony wall of the cochlea was perforated with a spherical drill head and the interior of the cochlea, including the spiral ganglion, was cleared. Cochlea and bulla were subsequently filled with gel foam and the wound was surgically closed. After a postoperative survival time of 3, 7, or 70 days (POD), the animals (11 controls, 4 at POD3, 11 at POD7 and 8 at POD70) received lethal doses of barbiturate (0.5 ml/200 g i.p., Narcoren, Merial GmbH, Hallbergmoos, Germany) and were transcardially perfused with a fixation solution containing 4% paraformaldehyde (PFA) and 0.1% glutaraldehyde in 0.1 M phosphate buffer (PB) at pH 7.4. Brains were removed from the skull and postfixed with 4% PFA in 0.1 M PB over night at 4°C. The cochlear nucleus was cut into 40 µm frontal sections on a microtome with vibrating blade (VT 1000, Leica, Bensheim, Germany).

### Tracer injection

For axonal tracing [21], rats (n = 3) received an intracochlear injection of biotinylated dextranamine (5–10% in distilled water, BDA, MW 10,000, Molecular Probes, Eugene, OR, USA). After surgical exposure of the cochlea, one or two small holes were drilled into its wall. Fifteen to 100 µl were injected in several portions through the holes. The cochlea was closed with bone wax and the wound treated as described. Injections were made 1 week before perfusion as above.

### EPTA staining

For ethanolic phosphotungstic acid (EPTA) staining [22], sections from healthy rats (n = 5) and from rats on which a unilateral cochlear ablation was done 3 (n = 3), 7 (n = 4), or 70 (n = 5) days before were dehydrated and stained by 1% phosphotungstic acid in 100% ethanol to which 2 drops (40 µl) of 96% ethanol per 10 ml solution were added for 1 h. Tissue from 3 healthy rats was processed for the simultaneous visualization of ultrastructure and EPTA staining by successive incubation in 1% osmium tetroxide (OsO_4_) for 30 min at room temperature (RT) in cacodylic buffer (CB), graded ethanol for dehydration, and 1% EPTA as above [23].

### Immuncytochemistry

For single immunostainings, free-floating sections were preincubated with 5% H_2_O_2_ for 30 min and 5% normal horse serum (NHS) in PBS. For the group of mouse antibodies, sections were then incubated with anti-synaptophysin (SyPhy) or anti-GAP-43 antibody (see Table 1) in PB with 0.8% NaCl (PBS) containing 2% NHS and 1% bovine albumin serum (BSA) for 72 h at 4°C, followed by treatment with the matching secondary antibody and Elite ABC (1∶250, Vector Laboratories, Burlingame, CA, USA) in PBS for 1 h at RT. For staining with rabbit anti-SyPhy 1 (Table 1), we used normal goat serum (NGS) and the biotinylated goat anti-rabbit IgG. Between every incubation step, sections were carefully rinsed in PBS. They were fixed in 1% glutaraldehyde in 0.1 M CB at pH 7.4. Peroxidase activity was visualized by incubation with 0.05% 3.3′-diaminobenzidine (DAB) and 0.0016% H_2_O_2_ in 0.05 M Tris-HCl buffer at pH 7.2 for 5 min. Sections were postfixed with 0.1% OsO_4_ in CB for 30 min, dehydrated, flat-embedded in EMbed-812 (Science Services, Munich, Germany) and mounted on glass slides. GAP-43 immunoreactivity covering the entire AVCN was used to verify completeness of cochlear ablation on the same side [24].

**Table 1:** Immunoreagents.

Antigen	Immunogene	Data	Dilution
Choline-Acetyltransferase (ChAT)	Human plazenta enzyme	Millipore (Temecula/CA, USA), goat polyclonal IgG, cat# AB144P	EM: 1∶500
Growth associated protein 43 (GAP-43) (clone 9-1E12)	43-48 kDa GAP-43 (B-50, F1 or pp46) purified from rat brain	Millipore, mouse monoclonal IgG_1_, cat# MAB347	LM: 1∶5,000, EM: 1∶1,000
Gephyrin (GlyR7a) (clone mAb7a)	N-terminus of purified rat gephyrin, 93 kDa splice variant	Synaptic Systems (Göttingen, Germany), mouse monoclonal IgG_1_, cat# 147 011	EM: 1∶200
Glutamate (clone GLU-4)	L-glutamic acid (Glu) (conjugated to KLH), reacts specifically with L-glutamate when conjugated	Sigma-Aldrich (Saint Louis/MO, USA), mouse monoclonal IgG_1_, cat# G9282	EM: 1∶1,000
Synaptophysin (SyPhy) (clone Sy-38)	Bovine 38-kDa integral membrane glycoprotein of the presynaptic vesicles	Boehringer Mannheim (Indianapolis/IL, USA), mouse monoclonal IgG_1_, cat# 902314	LM: 1∶100, EM: 1∶100
Synaptophysin 1 (SyPhy)	Synthetic peptide (GPQGAPTSFSNQM), corresponding to residues 301-313	Synaptic Systems, rabbit polyclonal serum, cat# 101 002	LM: 1∶200
Biotinylated horse, anti-mouse IgG	IgG (H+L) from mouse	Vector Laboratories Inc. (Burlingame/CA, USA), horse IgG, cat# BA-2001	LM: 1∶2,000 EM: 1∶2,000
Biotinylated rabbit, anti-goat IgG	IgG (H+L) from goat	Vector Laboratories Inc., rabbit IgG, cat# BA-5000	LM: 1∶2,000 EM: 1∶2,000
Biotinylated goat, anti-rabbit IgG	IgG (H+L) from rabbit	Vector Laboratories Inc., goat IgG, cat# BA-1000	LM: 1∶2,000 EM: 1∶2,000
Nanogold-anti-mouse IgG	IgG (whole molecule) from mouse	Nanoprobes (Yaphank/NY, USA), goat IgG, cat# 2001	EM: 1∶250
Nanogold-anti-goat IgG	IgG (whole molecule) from goat	Nanoprobes, rabbit Fab' fragment, cat# 2006	EM: 1∶250
Nanogold-streptavidin	——	Nanoprobes, cat# 2016	EM: 1∶100

LM: light microscopy; EM: electron microscopy.

For immunogold single stainings using antibodies of mouse origin (anti-SyPhy, anti-gephyrin, anti-GAP-43 and anti-glutamate, see Table 1), free-floating sections were preincubated with 5% NGS in PBS. The primary antibodies were diluted (see Table 1) in PBS-BSA-c (PBS with 0.1% acetylated BSA and 0.1% cold water fish skin gelatin, both from Aurion, Wageningen, Netherlands) with 1% NGS for 72 h at 4°C, followed by an interims incubation with 2% milk powder in PBS-BSA-c for 30 min and an incubation with anti-mouse antibody conjugated to Nanogold in PBS-BSA-c with 2% milk powder over night at RT. For the antibody of goat origin (anti-cholin acetyltransferase [ChAT]) we applied the matching normal serum and secondary antibodies. Between every incubation step we rinsed the sections carefully with PBS-BSA-c. The sections were fixed with 1% glutaraldehyde in CB for 10 min and gold particles were grown with a silver enhancement kit (HQ Silver, Nanoprobes, Yaphank, NY, USA) for 7–10 min. Some sections were fixed in 0.1% OsO_4_ for 20 min, dehydrated and flat embedded. From a couple of sections from each single immunogold staining we omitted the OsO_4_-incubation and treated them with EPTA as described above.

For double immunostaining, primary antibodies anti-ChAT and anti-SyPhy were supplied simultaneously in PBS-BSA-c for 72 h at 4°C, followed by incubation with biotinylated rabbit anti-goat in PBS for 1 h at RT and Nanogold-streptavidin in PBS-BSA-c with 2% milk powder over night at RT. Free streptavidin binding sites were blocked with 0.65 mM biotin (Sigma, Saint Louis, MO, USA) in PBS for 30 min. A biotinylated horse anti-mouse antibody in PBS was supplied for 1 h. After the gold particles were enhanced as described for single staining, sections were incubated in Elite-ABC for 1 h and peroxidase activity was visualized with DAB as described above. The procedure was completed by post-fixation, dehydration, and flat embedding. For each antibody and each experiment, staining controls were run by omitting the primary antibody and by mismatching the secondary antibody, requiring lack of staining in both cases.

For BDA-tracing, sections were incubated in Elite-ABC or Nanogold-streptavidin visualized with DAB or silver enhancement, respectively. Sections processed for immunogold staining were treated with EPTA. After hardening of the embedding resin, mounted sections were studied under the light microscope. Selected sections were reembedded in gelatin capsules and cut to semi-thin sections (0.5 µm) or ultra-thin sections (gold interference) on an ultramicrotome (Ultracut, Reichert-Jung, Vienna, Austria).

### Tissue shrinkage

Calculations presented here are anchored to the values calculated after ultrastructural analysis. Correction for shrinkage due to histochemical treatment was not made, so that all counts obtained from equally treated tissue are directly comparable in the present study.

Tissue shrinkage due to sensory deafferentation was assessed by comparing the area of AVCN on the affected side with the contralateral side. It was based on sections showing right-to-left or ipsilateral-to-contralateral AVCN profiles, of which the area was measured from controls (15 sections from 3 animals), POD7 (25 sections from 3 animals), and POD70 (27 sections from 3 animals), all at even spacing. Up to POD7, areal differences between sides were found to be non-significant. By POD70, cochlear ablation resulted in a highly significant (ANOVA, p<0.001) areal reduction of 0.66 on the affected side, or a shrinkage by 34%. No differences were encountered between AVCN section areas contralaterally at POD70 and age-matched controls. These results closely conform to those Benson et al. [25] obtained of AVCN in guinea pigs, who report 25% shrinkage by POD56 and 38% shrinkage by POD161.

If the tissue were mechanically unrestricted, areal shrinkage by 0.66 would correspond to volume shrinkage by [sqrt 0.66]^3^ = 0.53. However, VCN merges medially with other, non-auditory brainstem tissue. Therefore, shrinkage must be hampered in the dorsoventral and rostrocaudal dimensions so that volume shrinkage should fall short of arriving at this theoretical value. Consequently, the factor by which density values obtained in unit fields of tissue sections were multiplied to estimate changes in total number of counted items in tissue volume was rounded up to 0.6. We do not claim that tissue shrinkage, or processes of reorganization in general, are complete by POD70.

### Light microscopic evaluation

In a first step of quantitative assessment of profiles immunoreactive for SyPhy or GAP-43, semi-thin sections from DAB-immunostaining were used. Images (8-bit) were made with a x100 objective and a digital camera (Axiocam, Zeiss, Jena, Germany, connected to an Axiophot microscope, Zeiss). These photographs were imported into analySIS (Soft Imaging Systems, Münster, Germany) and computer-aided detection of immunoreactive elements was done by defining a detection threshold by reference to the mean gray value of a neutral zone which was kept constant for each type of antibody used. Detection was aided by defining a size window of 0.03 to 0.3 µm^2^ for profiles to be counted. Mean size of detection fields was 4,800 µm^2^.

### Electron microscopic evaluation

For the quantitative assessment of GAP-43, gephyrin and EPTA, ultra-thin sections from GAP-43 or gephyrin immunogold and EPTA staining or EPTA staining only were photographed on an electron microscope (Tecnai BioTWIN, FEI, Eindhoven, Netherlands) with a Megaview III digital camera (Soft Imaging Systems) at x11,000 magnification. After exposure to EPTA, selective electron opacification of tissue occurred at specific sites, while most other fine structural elements remain electron lucent. We counted all EPTA stained profiles per area and all profiles which showed 2 or more immunogold particles and EPTA precipitation simultaneously. Mean size of detection fields was 600 µm^2^.

Estimating the absolute density of synaptic contact zones (SCZs) identified by EPTA staining without making assumptions on their 3-dimensional shape, 20 pairs of ultra-thin sections were cut from embedded EPTA stained frontal sections of 3 brains. Within the core of AVCN with respect to all spatial dimensions, random fields were sampled to apply the physical dissector method [26]. Exactly the same procedure was applied to 20 pairs of frontal sections through the molecular layer of cerebellar flocculus, cutting this folded structure in all planes.

Values of absolute densities were determined in tissue from healthy animals and set to 100% (control levels). Lesion-dependent changes in tissue architecture were expressed in relative deviations from these control levels.

### Statistical analysis

We measured areal values, counted item densities and fractions, and calculated ipsilateral-to-contralateral ratios or total item number. For statistical testing, mean value and standard error of the mean (S.E.M.) were determined. The basis for statistical analysis of staining patterns in light microscopical sections is indicated for each histogram column in the respective Figures. Significant differences of counts were identified by applying one-way analysis of variance (ANOVA) followed by post testing (Newman-Keuls) with Prism (GraphPad Software Inc., LA Jolla, CA, USA). Significance levels were indicated as (***) for p<0.001, (**) for p<0.01 and (*) for p<0.05.

## Results

### Synaptophysin staining fails to label all presynaptic endings in AVCN

**Figure 1:**
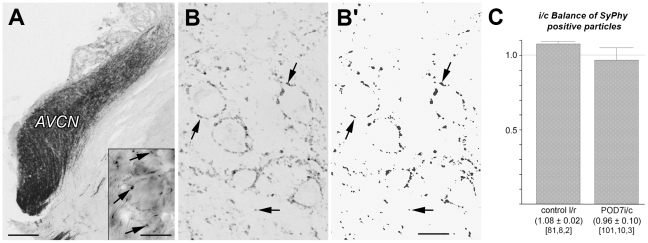
Anterograde tracing and SyPhy immunoreactivity in AVCN. (A) Anterograde axonal tracing with BDA from cochlea to AVCN, resulting in a great number of fiber segments and presynaptic endings darkly labeled (inset, arrows). Scale bar  = 200 µm, 20 µm for inset. (B) Pattern of SyPhy immunoreactivity and (B') rendering of staining for computer-based image analysis and counting of stained subcellular structures qualified by size and distribution to be presynaptic boutons (arrows). Scale bar  = 20 µm. (C) As must be expected, the number of such boutons is balanced between left and right side (control rat l/r) in normal hearing animals (left). Surprisingly, the balance persisted between ablated and unaffected side (right) by POD7, suggesting that SyPhy immunoreactivity does not reflect deafferentation-dependent changes taking place in the cochlear nucleus. Numeric triplets below columns indicate numbers of fields analyzed, tissue probes (embedded sections), and brains, respectively, on which statistical analysis is based.

The cochlear nerve covers the cochlear nucleus with a dense plexus of terminal arbors and presynaptic endings (Fig. 1A). To determine the fraction of synaptic contacts in the cochlear nucleus that are primary sensory afferents, we began collecting quantitative data on the density of synapses using 2 different antibodies raised against SyPhy, following previous studies [25], [27]. The antibodies were applied to tissue of AVCN from normal adult rats and from adult rats that had suffered unilateral cochlear ablation 7 days before. Based on the literature [14] we expected to observe a substantial decline of synapse number. Surprisingly, we failed to confirm this expectation with either of the two antibodies against SyPhy (Table 1). By statistical evaluation of SyPhy-positive profiles (Fig. 1B, B', C), the ratio between the stained profiles on the lesioned and unlesioned side, thought to represent presynaptic boutons, showed no significant decrease by POD7 (0.96±0.10 down from 1.08±0.02 in controls, Fig. 1C).

This finding leaves open whether SyPhy is an unreliable marker for presynaptic endings formed by spiral ganglion neurons or if primary afferents comprise a numerically negligible fraction in the neuronal network of the cochlear nucleus compared to afferents arising from sources other than the spiral ganglion. In order to test the dependability of SyPhy as a marker for presynaptic endings in AVCN, we double-stained spiral ganglion nerve endings by axonal tracing and SyPhy immunocytochemistry and analyzed them using electron microscopy.

**Figure 2:**
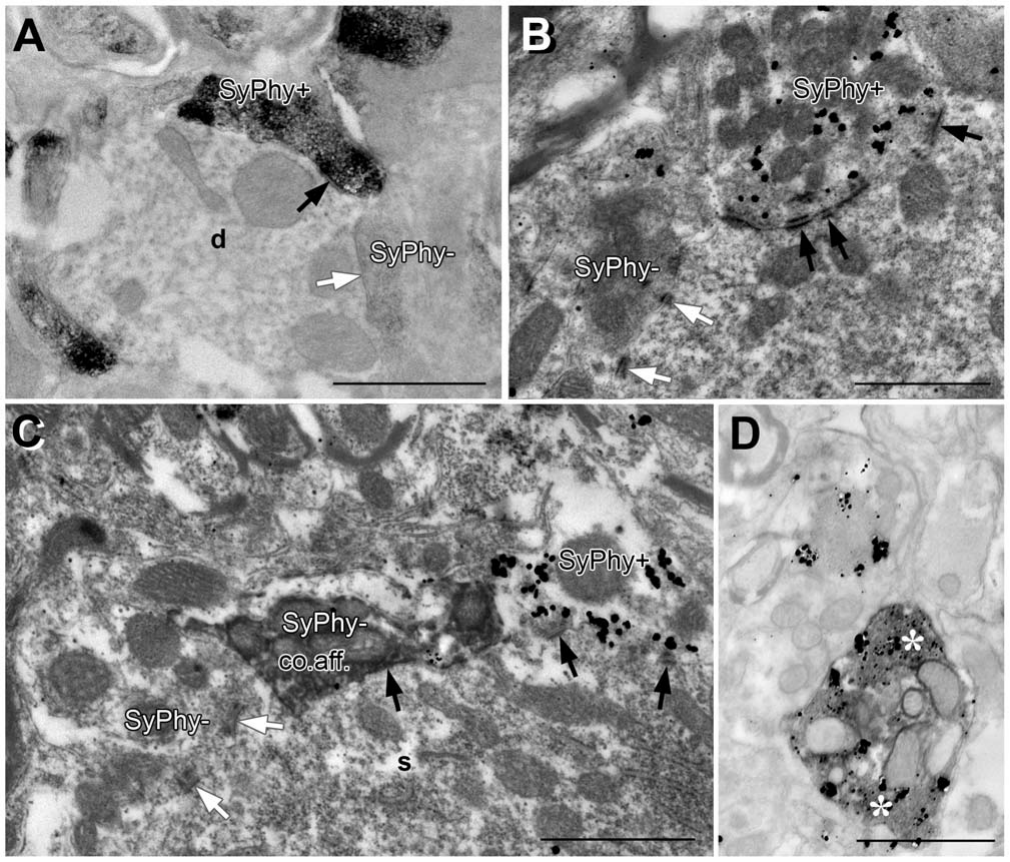
SyPhy immunoreactivity in AVCN detected under the electron microscope. (A) Staining with DAB already indicated that presynaptic profiles were not quantitatively recognized. Black arrows point to SCZs of immunopositive (SyPhy+) synapses, white arrows point to immunonegative (SyPhy-) synapses, always pointing from the postsynaptic side to an AZ; d  =  dendritic profile. (B) Immunogold staining (black particles) confirmed finding illustrated in A; same conventions and scale length. (C) Combining SyPhy immunolabeling with anterograde axonal transport of BDA from cochlear to AVCN proved that primary sensory synapses of various size do not dependably contain SyPhy, with nearby presynaptic profiles massively labeled for SyPhy through immunogold staining. Black arrows point to SCZs of a BDA-positive (co.aff.) or a SyPhy-positive synapses, white arrows point to an SCZ of a synapse negative for both BDA and SyPhy; s  =  neuronal soma. (D) Immunogold (SyPhy, black particles) and DAB (ChAT, white asterisks) double staining, indicating absence of interference between both procedures. Scale bar  = 1 µm for (A) – (D).

Synaptophysin was present in many presynaptic profiles, readily identified by presynaptic vesicles and membrane thickenings indicating SCZs (Fig. 2A, B). However, SyPhy immunoreactivity was not found in all BDA-labeled presynaptic profiles, with immuno-positive presynaptic profiles often immediately neighboring immuno-negative profiles (Fig. 2C). In particular, SyPhy was missing from many very large presynaptic endings known as endbulbs of Held [28]. Notably, SyPhy immunoreactivity was also absent from numerous presynaptic profiles that did not contain BDA and therefore may not belong to the primary sensory afferents. No methodological interference appeared to occur between immunogold and DAB staining (Fig. 2D).

As these findings clearly imply, synaptic terminals of spiral ganglion neurons do not consistently contain SyPhy, and can apparently operate without it. In order to discover what consequences a total auditory deafferentation has on the population of synapses in the cochlear nucleus, we had to discard SyPhy immunoreactivity as a suitable quantitative marker for presynaptic endings and look for an alternative.

### EPTA staining is a pan-synaptic marker

**Figure 3:**
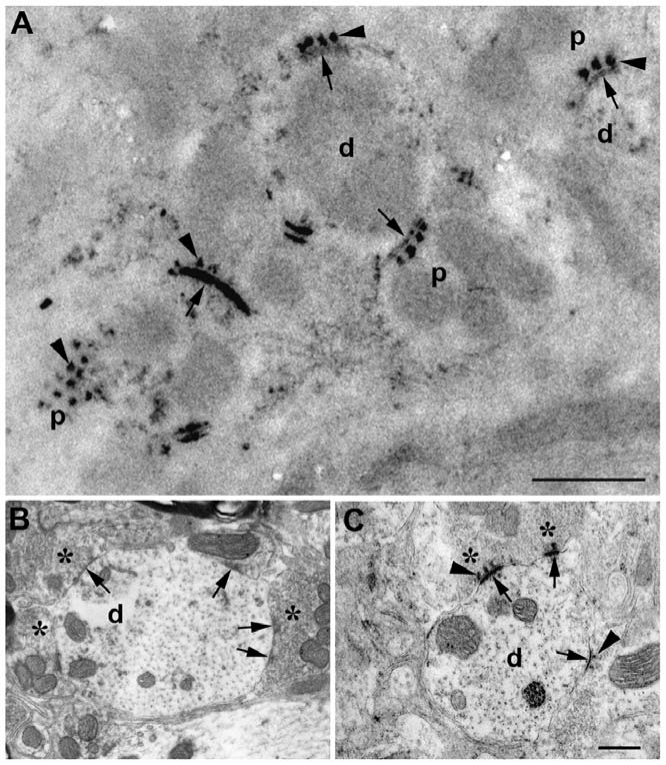
EPTA stained SCZs in AVCN of the normal adult rat. (A) Stained profiles appear dark against an otherwise unstained or grainy background and show, when cut perpendicularly, a characteristic morphology, including dense staining of the postsynaptic specialization, some of which marked by an arrow from the postsynaptic side, and, on the presynaptic side (p), ‘peaks’, ‘projections’, or ‘protrusions’ (arrowheads). When tangentially cut (lower left), these projections appear as a grid of patches. d  =  dendritic profile, scale bar  = 1 µm. (B) Section stained with OsO_4_ only, showing ultrastructural features such as synaptic vesicles (asterisks) and SCZs (arrows). (C) Section treated as in (B) but with an additional incubation with EPTA revealing presynaptic protrusions (arrowheads), representative of similar frames all failing to show SCZs stained by osmium only. Scale bar for B and C = 0.5 µm.

The EPTA treatment [22] visualizes SCZs, i.e. synaptic active zones combined with their postsynaptic counterpart, at high contrast (Fig. 3A). We also examined sections of AVCN treated for the simultaneous visualization of tissue ultrastructure and EPTA staining. Were EPTA not a pan-synaptic marker, we should have found synaptic contacts identified by a presynaptic vesicle pool associated to an interneuronal membrane apposition showing up as a thin osmium-typical darkening of the contact zone on both sides of the synaptic cleft without increased and extended contact zone display including presynaptic protrusions as is typical for EPTA staining (Fig. 3B). Screening 20 sections of 3 brains from normal adult rat, we failed to find any such instance (Fig. 3C). This confirms in double-stained material directly what Vrensen and De Groot [29] have shown by parallel section comparisons.

**Figure 4:**
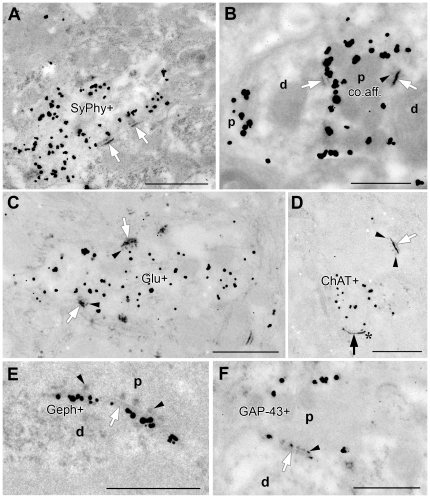
EPTA stains all types of SCZs. (A) EPTA staining does not interfere with immunogold detection, as shown here for SyPhy immunoreactivity, also proving that SyPhy positive synapses (SyPhy+) are readily recognized in EPTA treated material; arrows point to SCZs from the postsynaptic side. Scale bar  = 1 µm. (B) Unlike SyPhy immunoreactivity, EPTA does not fail to detect primary sensory endings (co.aff.) indicated by gold-detection of BDA transported into AVCN after intracochlear injection; p  =  presynapse, d  =  dendrite. Arrows point to SCZs from the postsynaptic side, arrowhead indicates presynaptic protrusions of an SCZ. Scale bar  = 0.5 µm. (C) Glutamate immunogold staining (Glu+) demonstrated in conjunction with EPTA at POD7, indicating that not all glutamatergic synapses have disappeared from AVCN after cochlear nerve degeneration. Arrows point to SCZ from the dendritic side, arrowheads indicate presynaptic protrusions. Scale bar  = 1 µm. (D) ChAT immunogold labeling (ChAT+) combined with EPTA staining at POD7; note that the ChAT-positive nerve ending carries an immature SCZ (asterisk), whereas a nearby ChAT-negative synaptic contact (white arrow) shows a fully differentiated EPTA staining including presynaptic protrusions (arrowheads). Scale bar  = 1 µm. (E) Gephyrin immunogold labeling (black particles, Geph+) combined with EPTA staining (dark gray). Arrow points to SCZ from the dendritic (d) side, arrowheads indicate presynaptic (p) protrusions. Scale bar  = 0.5 µm. (F) GAP-43 immunogold labeling (GAP-43+) combined with EPTA staining. Again, arrow points to SCZ from the dendritic (d) side, arrowhead indicates presynaptic (p) protrusions. Scale bar  = 0.5 µm.

We found additional evidence that EPTA is an indiscriminate and therefore quantitative marker for synaptic contacts in AVCN by testing various staining combinations. Apparently, EPTA labels SyPhy positive synapses (Fig. 4A), synapses prelabeled by BDA after transport from the cochlea (Fig. 4B), glutamatergic synapses (Fig. 4C) and cholinergic synapses identified by ChAT immunoreactivity (Fig. 4D). Similarly, EPTA stained SCZs were identified at inhibitory synapses marked by gephyrin (Fig. 4E) and at synapses labeled with GAP-43 (Fig. 4F). There was no indication that EPTA staining either failed to mark a specific type of synapse or that the EPTA method interfered with pre-embedding DAB or immunogold staining. Crucially, EPTA did not fail to capture primary afferents as SyPhy often did. These observations left us confident in the use of EPTA staining for the quantification of the number and density of SCZs in AVCN before and after total primary deafferentation.

### Absolute SCZ density

For the density of SCZs in fixed AVCN tissue of normal adult rats we calculated a value of 1.11±0.09×10^9^/mm^3^. Assuming the tissue exhibits an isotropic texture, we consider this an adequate estimate of contact zone density in AVCN. The density indicated does not include correction for shrinkage caused by the histochemical treatment. Changes against this value are expressed in relative deviations, observing counting criteria for EPTA stained profiles that were held unchanged for the material of control animals and all survival times.

For the purpose of control and comparison, we applied the identical protocol to determine the absolute density of SCZs in the molecular layer of the cerebellum. Our estimate of 2.32±0.12×10^9^ SCZs/mm^3^, or about twice as many SCZs as compared to AVCN neuropil, fell reasonably close to calculations made by other authors ([30]: 8×10^8^/mm^3^; [31]: 5×10^8^/mm^3^). These comparisons support our conviction that the method produces reasonable estimates.

### Sensory deafferentation causes a massive but incomplete synapse loss in AVCN

There were no changes in the density of EPTA stained SCZs in the AVCN on the side opposite to a unilateral cochlear ablation at any post-lesional time. Detecting EPTA stained SCZs in fields of up to 600 µm^2^ in sections of equal thickness (0.1 µm), we counted, normalized to 100 µm^2^, 8.17±0.24 in controls (49 fields from 5 brains), 9.42±0.38 by POD3 (29 fields from 3 brains), 8.34±0.34 by POD7 (39 fields from 4 brains), and 8.65±0.38 by POD70 (54 fields from 5 brains). Statistical analysis failed to reveal significant differences between these groups.

**Figure 5:**
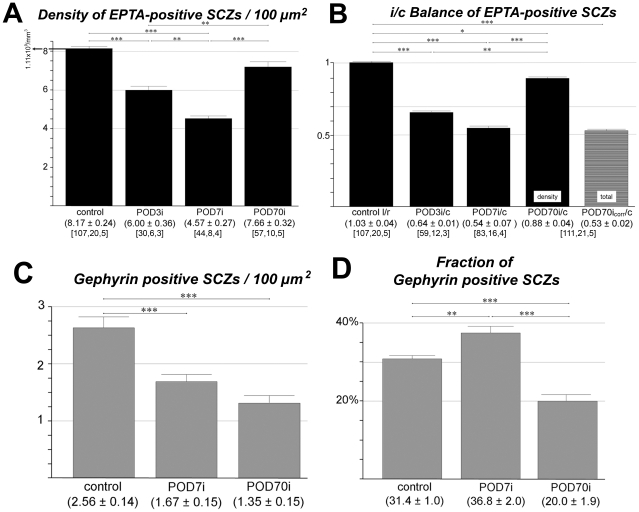
Quantification of EPTA profiles (all SCZs) and gephyrin positive profiles (inhibitory SCZs) before and at various times after cochlear ablation. (A) Progression of SCZ density after sensory deafferentation in the ipsilateral (i) AVCN. (B) Balance of ipsilateral-to-contralateral (i/c) ratio of EPTA stained SCZs; in controls left-to-right (l/r) ratios are given. When corrected for lesion-dependent tissue shrinkage (corr), the total number of SCZs present 10 weeks after cochlear ablation can be estimated (right column). (C) Density of gephyrin-positive profiles before and after cochlear ablation. (D) Fractions of gephyrin labeled SCZs among the population of all EPTA labeled SCZs present at pre- and postoperative stages. Numeric triplets below columns as in Fig. 1.

By sharp contrast, following a unilateral cochlear ablation 3, 7, or 70 days earlier, EPTA staining revealed a substantial and significant reduction of the density of SCZs on the side of the lesion (Fig. 5A, B). However, this reduction was far from exhaustive. A total sensory deafferentation of the cochlear nucleus caused a reduction to 54% of the original number of synaptic contacts that was still incomplete by POD3 but took full effect by POD7. After a post-lesional survival time of 10 weeks, the density of SCZs had almost fully recovered.

Whereas synapse density recovered, synapse number did not. We need to take tissue shrinkage into account to calculate the number of synapses remaining in AVCN after longer survival times following ipsilateral cochlear ablation. With an estimated average volume shrinkage of 0.6 by POD70 and an unchanged volume of the contralateral AVCN as judged by age-matched unablated animals, there was a reduction down to some 50% of the number of synapses in AVCN of normal animals (Fig. 5B), an observation fully compatible with the reported recovery of SCZ density.

### Excitatory and inhibitory synapses are differently affected by sensory deafferentation

Given the deafferentation-dependent reduction of synaptic number, the question arose whether excitatory and inhibitory synapses were equally or differentially affected in a dynamic reconstruction of the neuronal network or if the reduction of excitatory sensory synapses is stabilized over time. We sought an answer to this question using gephyrin immunoreactivity as a marker for SCZs of inhibitory synapses [32], [33] before and after cochlear lesion.

We found that the density of gephyrin positive profiles in the ipsilateral AVCN differed significantly between control level and POD7, being reduced by 30% (Fig. 5C). As there is no tissue shrinkage by this survival time, it can be concluded that the total number of inhibitory synapses also decreased within one week of ablation. Obviously, then, sensory deafferentation has a quick effect on the population of inhibitory synapses as well as on excitatory synapses. This suggests that by this stage of neuroplastic response to sensory deafferentation, the ratio of inhibitory to excitatory synaptic contacts has changed in favor of excitatory synapses. To settle this issue, we determined the ratio of gephyrin-positive SCZs to all SCZs, the latter being determined by EPTA staining.

In AVCN of the normal adult rat, 31.4±1.0%, or about one third, of all SCZs were gephyrin-positive and therefore inhibitory (Fig. 5D). There was a significant increase in the fraction of inhibitory synaptic contacts by POD7, suggesting a transient over-inhibition in AVCN on the affected side (Fig. 5D). From POD7 to POD70 this ratio strongly turned in the other direction, now indicating a relative lack of inhibitory transmission (Fig. 5D). Correcting for tissue shrinkage revealed that the total number of inhibitory SCZs decreased to a third of the normal value. At the same time, the number of all synapses has shrunk to just above half of the original level. Correspondingly, we determined a fraction of inhibitory SCZs of only 20.0±1.9% by POD70. Together these numbers suggest a potential over-excitation in the interneuronal communication inside AVCN as the eventual result of a total unilateral deafferentation.

The density of gephyrin positive SCZs in the AVCN contralateral to sensory deafferentation per 100 µm^2^ was 1.94±0.13 by POD7 (37 fields from 3 brains), and 2.15±0.22 by POD70 (33 fields from 3 brains). Compared to unablated controls (2.56±0.14, 65 fields from 3 brains) there was a marginal significant decrease (p<0.05) by POD7 but not by POD70.

### Sensory deafferentation causes emergence of GAP-43 positive nascent synapses

**Figure 6:**
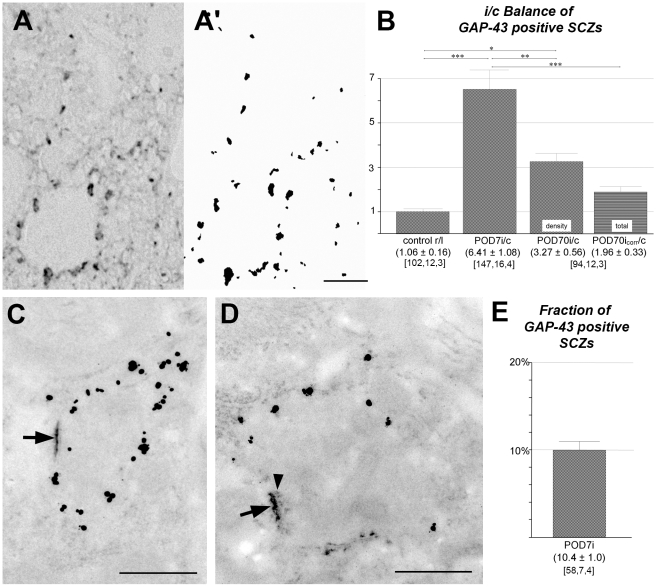
Quantification and ultrastructure of GAP-43 positive profiles in AVCN. (A) Semi-thin section stained for GAP-43 immunoreactivity. (A') Frame shown in A rendered for computer-based image analysis and quantification. Scale bar  = 20 µm. (B) Ipsilateral-to-contralateral ratio of GAP-43 positive profiles in controls and after 7 and 70 days of cochlear ablation. The right-most column shows counts after correction (corr) for tissue shrinkage to indicate the relative total of GAP-43 positive profiles present by POD70. (C) Ultrastructure of a synaptic contact by POD7 showing rich immunogold staining for GAP-43 typically located inside the presynaptic ending, avoiding occupation of SCZs which is here distinctly immature as revealed by EPTA staining (arrow). Scale bar  = 0.5 µm. (D) Another synapse present by POD7 with a more mature contact zone (arrow, arrowhead points to row of presynaptic protrusions) and correspondingly less GAP-43 immunogold labeling. Scale bar  = 0.5 µm. (E) Fraction of GAP-43 positive synaptic contacts among all SCZs present by POD7 on the lesioned side (i). Numeric triplets below columns as in Fig. 1.

Based on staining for GAP-43, we sought to evaluate the density and number of nascent synaptic profiles before and after unilateral cochlear ablation. Sections through AVCN were stained for GAP-43 immunoreactivity (Fig. 6A). From these, semi-thin sections were cut, the pattern of staining photographed, transformed into quantifiable pictures (Fig. 6A'), and analyzed for size and number of stained profiles in normal rats and at 7 and 70 days post-lesion. Contralaterally, GAP-43 immunoreactivity remained at low control level through all stages. Ipsilaterally, the number of particles varied greatly with post-lesional time (Fig. 6B). The ratio of ipsilateral-to-contralateral particle number rose from 1∶1 to 7∶1 by POD7, indicating a massive emergence of GAP-43 positive detected profiles on the side of sensory deafferentation. The subsequent slow collapse of this asymmetry is due to a decline of GAP-43 immunoreactivity on the affected side rather than a gain in the contralateral AVCN.

The formation and maturation of an SCZ can be seen by EPTA staining to pass through several stages, ranging from a mere hint of membrane opacification to a fully developed bilaminar staining with prominent protrusions that extend into the lumen of the presynaptic cell [34]-[37]. When GAP-43 immunolabeling and EPTA staining were applied to the same section, GAP-43 immunoreactivity was typically found inside presynaptic endings and close to its outer membrane, but outside the contact zone (Fig. 6C, D). Remarkably, there appeared to be an inverse relation between the amount of immunogold particles and the maturational status of the contact zone. In presynaptic endings with strong GAP-43 labeling, SCZs were typically ill-differentiated and pale. With decreasing intensity of GAP-43 labeling we saw a tendency for SCZs to be better differentiated in two bands, with pale to distinct presynaptic protrusions. Double-stained ultra-thin sections were quantitatively analyzed by counting EPTA-positive SCZs bare of, or combined with, GAP-43 immunoreactivity. At the maximum of GAP-43 staining by POD7 [20], SCZs unequivocally associated with nearby GAP-43 immunoreactivity made up a fraction of 10.4±1.0% from all SCZs (Fig. 6E).

Contralateral to cochlear ablation, GAP-43 immunoreactivity remained at a barely detectable level through all stages studied. The density of immunoreactive profiles normalized to a test field size of 100 µm^2^ was 0.53±0.03 in normal rats (102 fields from 3 brains), 0.77±0.07 by POD7 (73 fields from 4 brains), and 0.80±0.10 by POD70 (49 fields from 3 brains). All counts were close to the detection threshold so that debating significant differences among them would appear to be venturesome.

## Discussion

Primary sensory afferents form less than half of all SCZs in VCN (Fig. 5A). Given that these afferents include calyciform endings that contain on average over 150 SCZs each [38], this fraction decreases even more when the number of axonal endings is considered. Apparently, an extended neuronal network remains in AVCN even in the absence of direct sensory input. Although this network is immediately silenced after acute cochlear ablation [17], development of cellular processes was initiated to mould a modified network architecture within weeks.

### Presynaptic endings vs. SCZs

Unlike molecular markers indicating presynaptic endings such as SyPhy, ChAT, or GAP-43, EPTA staining and gephyrin immunoreactivity depict single SCZs. Whereas a synapse comprises one presynaptic ending, it may have more than one contact zone, a structurally specialized region serving presynaptic vesicle fusion and postsynaptic transmitter reception [39]. In a nerve net, the density of presynaptic endings more closely reflects the potential for external sources to control network activity, whereas the density of contact zones rather reflects the postsynaptic activity level induced by a given number of presynaptic endings.

### All vs. inhibitory SCZs

Adult cochlear nerve axons release an amino acid neurotransmitter, probably glutamate, at their terminals to induce excitatory action at their postsynaptic target cells [40]–[42]. Consequently, disconnecting cochlear nerve axons from their parent cells will not axotomize inhibitory neurons. The question therefore arose whether there is a response to sensory deafferentation of the extensive population of inhibitory synaptic contacts present in the cochlear nucleus, and, if so, what extent it would assume and in what direction this might go. We found that the inhibitory nerve endings responded with synapse elimination to the axotomy affecting other neurons surprisingly quickly. When the total of all SCZs has dropped to about half of their original number by POD7 (Fig. 5A), the inhibitory synaptic contacts have decreased by only one third (Fig. 5C). These changes mean that the fraction of inhibitory synapses among all synapses must have increased, at least transiently (Fig. 5D).

Following this state of potential over-inhibition on the deafferented side of AVCN by POD7, the density of inhibitory SCZs further declined against an unchanged level of total SCZs (Fig. 5D). Toward POD70, the fraction of gephyrin-positive SCZs fell below their original fraction, such that excess excitation occurred in the neuronal network. Whatever signals are fed into AVCN deprived from direct sensory input now appears to have an increased likelihood to generate excitation spreading along the ascending auditory system. Potentially, this might generate a source for tinnitus.

### Nascent synapses

In the normal adult rat, neurons of the ventral nucleus of the trapezoid body (VNTB) giving rise to the medial olivocochlear projection send axon collaterals into AVCN [6], [43], [44]. If this nucleus is destroyed contralaterally or bilaterally before sensory deafferentation, GAP-43 positive boutons fail to show up in AVCN on the side of cochlear ablation [45]. What we see as GAP-43 positive profiles in AVCN after cochlear ablation is supplied by cholinergic VNTB neurons, as all GAP-43 positive presynaptic endings present in AVCN are cholinergic [46]. Apparently, then, it is not re-innervation from other sensory systems, such as close-by vestibular or somatosensory regions, that we encounter here, but an intra-auditory synaptic replacement. Some synaptic profiles described in earlier ultrastructural studies must correspond to these GAP-43 positive elements. As patterns of ‘clear-type degeneration’ [14] or ‘flocculent degeneration’ [2] were described as untypical, it may be worthwhile to reconsider them as including sprouting axons and their terminals.

At GAP-43 peak expression by POD7 [20], presynaptic endings rich in GAP-43 tend to show immature SCZs as revealed by EPTA staining (Fig. 6C). When GAP-43 expression fades away, associated SCZ morphology tended to be more maturely elaborated (Fig. 6D). This suggests that the transient emergence of GAP-43 observed does indeed indicate formation and maturation of synaptic contacts, a result fully consistent with the emergence of EPTA stained SCZs in the moment of developmental onset of synaptic transmission [47].

With every tenth SCZ associated with GAP-43 immunoreactivity in the time window open at POD7 (Fig. 6E), this must be a low border of the true fraction of new synaptic contacts added over the entire post-lesional reorganization for three reasons. Due to the spatial distance between EPTA staining at the synaptic contact zone and GAP-43 immunoreactive sites at the side or in the shaft of presynaptic boutons, their spatial association is easily missed when examining ultra-thin sections that must in many cases have been cut between them. This argument does not apply to the quantitative identification of gephyrin-EPTA double labeling, both manifest on sub-cellular elements in immediate vicinity at the contact zone (Fig. 4E). Moreover, we can be certain about a temporal overlap but not about a complete temporal coincidence of EPTA and GAP-43 staining. For instance, SCZs may not yet be stainable by EPTA at early stages of synaptogenesis when GAP-43 staining is already high. Finally, since some axons may grow faster and arrive earlier in AVCN than others, it remains doubtful if the entire population of boutons carrying GAP-43 is present and detectable at any single point in time.

From POD7 to POD70, 17% of all SCZs disappeared; these were gephyrin positive (Fig. 5D). They had to be replaced to comply with an unchanged level of total SCZs (Fig. 5B). These facts should rebut a view according to which GAP-43 positive fibers and boutons emerge at some post-lesional point in time but disappear again with vanishing GAP-43 immunoreactivity.

Long-lasting nervous structural formations related to GAP-43 have been an issue in studies on the development of diverse cerebral systems. The formation of orderly connected axons apparently requires GAP-43, as proper axon routing fails in GAP-43 knockout mice in the visual [48] and the somatosensory system [49]. Deafferentation-induced cortical plasticity has been shown in adult rats following vibrissectomy [50], leaving open whether changes in GAP-43 staining indicate movement of axons, their sprouting, or synapse formation. Interestingly, GAP-43 immunoreactivity is retained in specific corticothalamic and corticotectal fibers of the adult rat, taken to indicate ongoing neuroplastic dynamics [51]. Unlike the GAP-43 terminals described in the present study which we know contain ChAT and therefore appear to be cholinergic [46], at least some of the corticofugal fibers contain GABA [51]. This suggests that GAP-43 may identify dynamic axons independent of their transmitter content and polarity of action.

### Deafferentation-induced dynamic network response

There appears to be a complex structural dynamic in the adult cochlear nucleus induced by sensory deafferentation which we attempt to reconstruct as follows.

**Figure 7:**
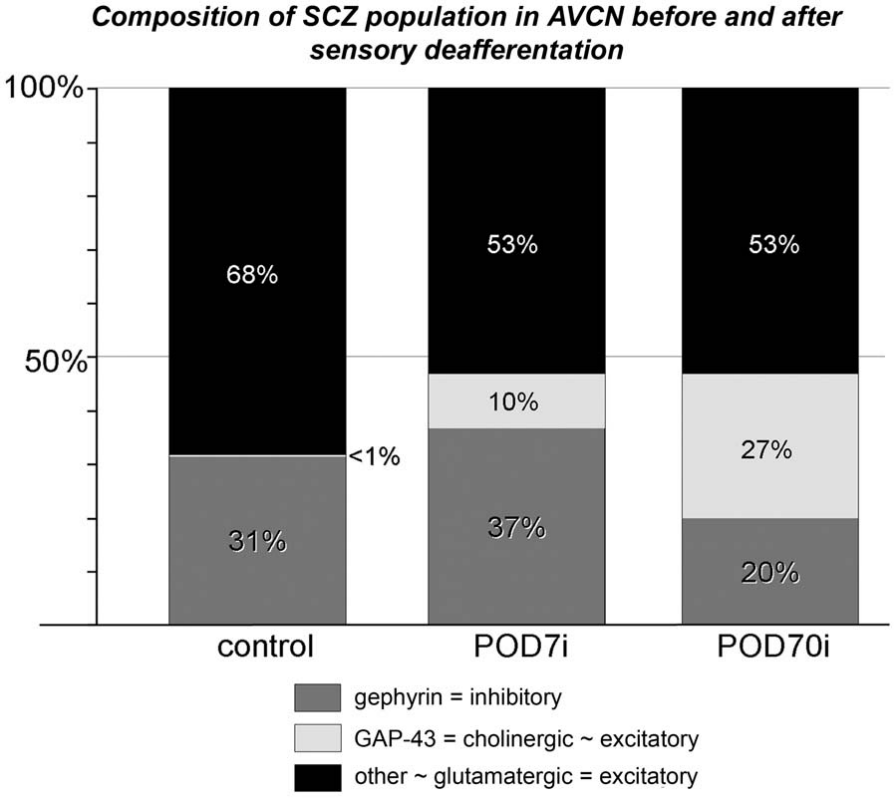
Composition of the population of SCZs in adult controls, by 7 and by 70 days following cochlear ablation. A deafferentation induced reorganization of the neuronal network in AVCN includes a transient rise of inhibitory synapses and the emergence of GAP-43 positive synaptic contacts. After 10 weeks excitatory synaptic contacts have increased in relative number, apparently due to an extended fraction of cholinergic synapses. ‘∼’ translates to ‘suspected to be’.

1. All evidence available suggests that EPTA stains all types of SCZs, rendering it a quantitative marker for synaptic contacts in AVCN (Fig. 4A–F).
2. Relative changes of the total number of EPTA stained profiles determined in AVCN by 3, 7, and 70 days after total cochlear ablation on the side of the lesion were found to have declined by POD7 by 46% and stayed at this level until POD70, while SAZ density recovered by POD70 to an almost normal level (Fig. 5A).
3. The fraction of gephyrin-positive profiles identifying inhibitory synapses among the entire synaptic population was determined in normal animals to be 31%, rising to 37% by POD7 (Fig. 5D). Had the number of gephyrin synapses remained unchanged while the total synaptic population was reduced to 54% (Fig. 5B), the fraction of gephyrin synapses should have risen to 57%, which it failed to do. Indeed, a reduction of gephyrin synapses on the deafferented side down to 62% of control levels was noticed by POD7 (Fig. 5C). Apparently, there is an ablation-induced decline of inhibitory synapses that begins almost immediately after sensory deafferentation but trails the loss of excitatory synapses. This delay in the decline of inhibition may lead to a transient over-inhibition in the neuronal networks of AVCN.
4. The decline of inhibitory synapses continues beyond POD7. By POD70, only 20% of all SCZs then present were gephyrin-positive (Fig. 5D). The numerical relationship between excitatory and inhibitory synaptic contacts in the normal adult AVCN (2.2∶1) appears to shift to a transient and relative over-inhibition by POD7 (1.7∶1), only to turn to potential over-excitation by POD70 (4∶1). Obviously, this must have consequences for signal processing of neuronal networks in AVCN and beyond.
5. With essentially all primary afferents being lost by POD7 (Fig. 5A, B; cp. also [14]), a constant number of SCZs persisted thereafter (Fig. 5B). As a significant and ongoing decrease of gephyrin-positive synapses (Fig. 5C) took place, new excitatory synapses must have filled the gap. Indeed, presynaptic endings containing GAP-43 massively increase in number after cochlear ablation from a marginal level in the normal adult AVCN (Fig. 6B), all of them containing ChAT [46], likely to indicate excitatory cholinergic synapses [52]. Eventually, the post-deafferentation network in AVCN should therefore comprise at least 27%, or close to one third, of newly formed synapses (Fig. 7). This suggests a high constitutive intrinsic potential for network reorganization of VCN.

### Adult synaptogenesis

With the exception of olfactory bulb and hippocampal formation [53], adult neurogenesis seems to be rare at best. Therefore, adult synaptogenesis must be preceded by axonal growth from pre-existing neurons. In such cases, homotypical re-generation needs to be distinguished from heterotypical re-innervation. If neurons lose afferent synaptic contacts as a consequence of brain lesion or dysfunction, a re-occupation of the abandoned postsynaptic sites may be accomplished by axons growing out from sources equivalent to the lesioned one, such as neighboring cortical regions. This would result in a homotypic re-generation of synaptic contacts as seen in the hippocampus after cortical lesions [54] or on corticospinal neurons after spinal cord injury [55]. At least the latter is certain to result in a convincing regain of function.

If no equivalent to the lesioned nerve or brain region is available to serve re-occupation of a denervated target, then re-innervation, if occurring, must be supplied by a different, or heterotypic, part of the brain. A total cochlear ablation as done in the present study rules out homotopic re-generation. However, axonal growth and synapse formation described here are accomplished by neurons already being connected to the cochlear nucleus by axon collaterals in the normal brain [45]. Cutting the peripheral axonal branch of these cells that normally reaches the inner ear to innervate outer hair cells in the course of cochlear ablation may contribute to turn the neurons into a sprouting mode.

There is evidence that the re-innervation of the cochlear nucleus following the total loss of one ear is functionally meaningful. Kraus et al. [56] suggest that synaptogenesis in the cochlear nucleus as indicated by the temporary rise of GAP-43 serves to suppress tinnitus in adult rats that arose as a result of unilateral noise trauma.

It is expected that adult synaptogenesis requires the action of a variety of growth-related molecules [57]–[59] and the participation of astroglial cells [60], [61]. However, to find that the heterotypic re-innervation we describe closely matches in time course and breadth the homotopic re-generation in an entirely different system [54] suggests that the signals initiating re-innervation and synaptogenesis may not be strictly discriminative with respect to the particular type and molecular profile of growth cones that are accepted to approach. Occurrence and volume of synaptogenesis may be determined by a setting of the neuropil characterized by synaptic packing density and vacant, i.e. mechanically accessible, sites that invites nerve fibers capable of sprouting indiscriminately of their origin.
